# The Global Gap in the Hemophilia Paradigm Shift: Disparities in Research, Care, and Musculoskeletal Health

**DOI:** 10.3390/hematolrep18030042

**Published:** 2026-06-22

**Authors:** Felipe Querol-Giner, Magdalena Querol-Giner, Ana Chimeno-Hernández, Pilar Alberola-Zorrilla, Sofía Pérez-Alenda, Santiago Bonanad, Felipe Querol-Fuentes

**Affiliations:** 1 PTinMotion Research Group, Department of Physiotherapy, University of Valencia, 46010 Valencia, Spain; felipe.querol-giner@uv.es (F.Q.-G.); sofia.perez-alenda@uv.es (S.P.-A.); 2 Department of Rehabilitation, Hospital Clínico Universitario de Valencia, 46010 Valencia, Spain; 3 Department of Physiotherapy, University of Valencia, 46010 Valencia, Spain; 4 Hemophilia Association of the Valencian Community (ASHECOVA), 46018 Valencia, Spain; pilar.alberola@uv.es; 5 Department of Anatomy and Human Embryology, University of Valencia, 46010 Valencia, Spain; 6 Coagulation Disorders Unit, Department of Hematology, Hospital Universitario y Politécnico La Fe, 46026 Valencia, Spain

**Keywords:** hemophilia, global disparities, therapeutic advances, emicizumab, gene therapy, physiotherapy, musculoskeletal ultrasound, hemophilic arthropathy

## Abstract

**Background:** Hemophilia care has undergone a major therapeutic transformation with the introduction of extended half-life products, non-replacement therapies, and gene therapy. However, the benefits of these advances are not equally distributed worldwide, and their impact on long-term musculoskeletal outcomes remains uncertain. **Objective:** To analyze global disparities in hemophilia care and research production in the context of recent therapeutic advances, with particular attention to musculoskeletal management, physiotherapy, and scalable strategies for resource-limited settings. **Methods:** A narrative review with a structured literature search was conducted. Two conceptual blocks were explored: global disparities and access to care in hemophilia, and recent therapeutic advances, including non-replacement therapies, extended half-life products, and gene therapy. Retrieved records were screened using Rayyan, and a structured workflow diagram was used to summarize the literature identification and selection process. A descriptive analysis was also performed to identify representative authors, institutions, and geographic patterns in hemophilia research. **Results:** The evidence shows substantial global disparities in diagnosis, access to treatment, healthcare infrastructure, and research production. Scientific output remains concentrated in high-income countries, while low- and middle-income regions are underrepresented. Advanced therapies consistently reduce bleeding rates and treatment burden, but concerns remain regarding access, affordability, durability, breakthrough bleeding, and long-term structural joint outcomes. Musculoskeletal complications, including subclinical bleeding and hemophilic arthropathy, remain clinically relevant despite improved hematologic control. **Conclusions:** The current paradigm shift in hemophilia care is not uniformly experienced worldwide. Addressing global disparities requires not only expanding access to advanced therapies, but also strengthening research capacity, implementing multidisciplinary care models, and integrating scalable interventions such as physiotherapy, patient education, and simplified diagnostic tools. Accessible musculoskeletal assessment strategies may help improve early detection, functional outcomes, and equity of care in resource-limited settings.

## 1. Introduction

Hemophilia is a rare inherited bleeding disorder characterized by recurrent bleeding episodes, particularly affecting the musculoskeletal system, leading to progressive joint damage and disability [1]. According to the World Health Organization (WHO), rare diseases collectively affect more than 300 million people worldwide and represent an increasing global public health challenge [2]. Hemophilia is considered a rare disease, with an estimated prevalence of approximately 17.1 cases per 100,000 males for all severities and 6.0 cases per 100,000 males for severe hemophilia [3]. Despite significant advances in pharmacological treatment, including prophylaxis and novel therapies, access to these interventions remains highly unequal worldwide.

In recent years, hemophilia care has undergone a profound transformation, driven by the development of extended half-life factor concentrates, non-replacement therapies such as emicizumab, and the emergence of gene therapy. Gene therapy approaches aim to achieve sustained endogenous clotting factor expression, potentially reducing bleeding frequency and the need for regular prophylactic replacement therapy. These advances have significantly reduced bleeding rates and have redefined expectations regarding disease control and long-term outcomes, representing a true paradigm shift in hemophilia management [4,5].

It is estimated that a substantial proportion of individuals with hemophilia globally do not receive adequate treatment, particularly in low- and middle-income countries (LMICs), where access to clotting factor concentrates is limited or absent [6,7,8]. While this gap has been widely acknowledged, less attention has been paid to disparities in access to non-pharmacological interventions, such as physiotherapy, which are essential for the prevention and management of musculoskeletal complications [9].

In parallel, the scientific literature on hemophilia is largely dominated by high-income countries with well-established healthcare and research infrastructures. Countries such as Canada, the United States, and several European nations contribute disproportionately to the global body of evidence, often through integrated academic-clinical networks that facilitate large-scale data collection and analysis [3,10].

However, this concentration of knowledge production raises concerns regarding the generalizability and applicability of current clinical evidence in resource-limited settings. The clinical realities of patients in LMICs—characterized by delayed diagnosis, lack of prophylaxis, and advanced joint disease—are often underrepresented in the literature [11].

As a consequence, a mismatch becomes evident between therapeutic progress and its translation into routine clinical practice across different healthcare settings. This highlights that the current paradigm shift in hemophilia care is not uniformly experienced worldwide [6].

Moreover, while pharmacological advancements continue to evolve, including the development of longer-acting therapies, non-replacement treatments, and gene therapy approaches aimed at improving bleeding control and reducing treatment burden, complementary non-pharmacological interventions remain important. In this context, physiotherapy and simplified diagnostic approaches, including focused musculoskeletal assessment strategies, contribute to improving functional outcomes and long-term joint management in settings where advanced therapies are not accessible [12,13].

Therefore, the objectives of this study are:

1. To map the academic institutions and authors contributing to hemophilia research;
2. To identify global disparities in knowledge production and access to care, particularly in the context of recent therapeutic advances;
3. To analyze the implications of the current paradigm shift in hemophilia treatment across different healthcare settings;
4. To discuss the role of physiotherapy, simplified diagnostic approaches, and context-adapted complementary care strategies for resource-limited settings.

## 2. Materials and Methods

A narrative review with a structured and reproducible search strategy was conducted to explore global disparities in hemophilia care and the impact of recent therapeutic advances. The literature search was intended to support and contextualize the narrative framework of the review rather than to provide an exhaustive systematic assessment of all available evidence. Accordingly, the review combined a targeted literature search with a narrative synthesis of the available evidence, allowing contextual interpretation of global disparities and therapeutic advances in hemophilia, including discussion of published bibliometric studies and global epidemiological data related to hemophilia research and healthcare infrastructure, incorporated as complementary contextual elements within the narrative framework of the review.

The literature search was organized into two conceptual blocks: (1) global disparities and access to care in hemophilia, and (2) recent therapeutic advances, including non-replacement therapies, extended half-life products, and gene therapy.

For the disparities block, the following search equation was applied in PubMed:

(hemophilia OR haemophilia) AND (“health disparities”[Title/Abstract] OR “access to care”[Title/Abstract] OR inequity[Title/Abstract])

Filters were applied to include studies published within the last five years, conducted in humans, and written in English or Spanish.

For the disparities block, studies were included if they addressed global inequities, access to care, or differences between high-income and low- and middle-income countries (LMICs) in hemophilia. Eligible studies also included those describing availability, access, or limitations of treatment, as well as research adopting a population-level, healthcare system, or global health perspective.

Studies were excluded if they were not related to hemophilia, focused exclusively on clinical efficacy without addressing disparities or access, or consisted of case reports, small case series, or purely technical studies. Conference abstracts without full text or insufficient data were also excluded.

For the therapeutic advances block, the search equation used was:

(hemophilia[Title/Abstract] OR haemophilia[Title/Abstract]) AND (emicizumab[Title/Abstract] OR “extended half-life”[Title/Abstract] OR “gene therapy”[Title/Abstract])

To enhance methodological consistency and prioritize higher levels of evidence, this search was restricted to systematic reviews. The same filters (last five years, humans, English or Spanish) were applied.

For the therapeutic advances block, studies were included if they addressed recent advances in hemophilia treatment, including non-replacement therapies, extended half-life products, or gene therapy. Eligible studies were required to report clinically relevant outcomes, such as reduction in bleeding rates, improvement in quality of life, or overall clinical impact. Only systematic reviews were included, in order to prioritize higher levels of evidence and ensure methodological consistency.

Studies were excluded if they focused exclusively on pharmacokinetics, dosing, or molecular mechanisms, or if they consisted of randomized clinical trials without broader synthesis or interpretation. Case reports, small case series, and studies not addressing clinical outcomes or real-world impact were also excluded.

All retrieved records were exported to Rayyan (QCRI, https://rayyan.ai) for screening and selection. Duplicate records were identified and removed prior to screening. A two-stage screening process was performed, consisting of title/abstract screening followed by full-text evaluation, which led to the exclusion of a small number of studies not meeting the predefined criteria.

Screening decisions were based on predefined exclusion criteria and standardized labels applied within Rayyan, adapted according to the objectives of each conceptual search block. The final selection was based on relevance, methodological clarity, and contribution to the study objectives.

**Figure 1:**
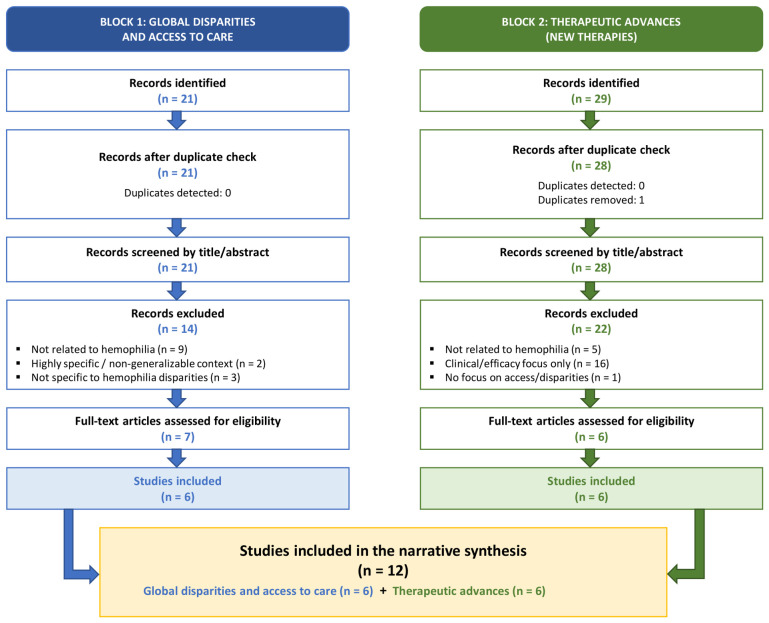
Structured workflow illustrating the literature identification and selection process across the two predefined conceptual search blocks.

The literature identification and selection process is summarized in a structured workflow diagram (Figure 1). The workflow diagram was included to improve transparency in the literature identification and selection process within the narrative framework of the review. The diagram is therefore used solely as a structured representation of the screening process.

In addition to the structured search, a descriptive analysis was performed to identify key authors, institutions, and geographic patterns in hemophilia research, with the aim of contextualizing global disparities in knowledge production.

## 3. Results

### 3.1. Disparities in Hemophilia Care

The analysis included 12 studies addressing both global disparities and recent therapeutic advances in hemophilia (Table 1). The included literature encompassed a wide range of study populations, including pediatric patients, women and girls with bleeding disorders, inhibitor patients, and large multinational cohorts, reflecting the heterogeneous clinical and healthcare contexts associated with hemophilia management worldwide.

From a global perspective, substantial disparities in access to diagnosis, treatment, and healthcare infrastructure were consistently reported, particularly in low- and middle-income countries [7,9,14]. These disparities were further exacerbated in specific populations such as women, who remain underdiagnosed and undertreated [15,16].

Several studies suggest that these disparities are driven by structural barriers related to healthcare organization, resource availability, diagnostic capacity, and access to specialized care [7,9,14,15,16,17].

Similarly, all included studies addressing disparities consistently reported structural inequities in access to care and healthcare systems [7,9,14,15,16,17].

**Table 1:** Summary of selected studies on disparities and therapeutic advances in hemophilia.

Author, Year	Study Type	Population/Sample	Key Findings
Coffin et al., 2026 [9]	Global survey (WFH)	>70 countries (NMOs)	Limited access, underdiagnosis, major service gaps (especially in women).
Majda et al., 2025 [17]	Population model	~6512 patients (USA)	Emicizumab improves outcomes and may reduce inequities; system-dependent.
Hermans et al., 2024 [15]	Narrative review	Not applicable	New therapies benefit mainly males; women underdiagnosed/undertreated.
Jena et al., 2025 [14]	Narrative review	Not applicable	Persistent unmet needs: limited access, ongoing bleeding, arthropathy risk.
Arya et al., 2021 [16]	Qualitative study	15 women (Canada)	Barriers: poor recognition, limited access, lack of specialist care.
Laliberté et al., 2023 [7]	Strategic report	>200 stakeholders, 70 countries	>70% lack access; advanced therapies may widen disparities.
Olasupo et al., 2026 [4]	Cochrane review	397 patients	Non-replacement therapies ↓ ABR, ↑ QoL; limited long-term/structural data.
Deshpande et al., 2024 [5]	Meta-analysis	395 patients (HA + HB)	Gene therapy ↓ bleeding and factor use; durability uncertain.
Silva Mendes et al., 2026 [18]	Meta-analysis	5 studies	Gene therapy ↓ ABR and treatment burden; some require reintroduction.
Han et al., 2024 [19]	Meta-analysis	11 trials	Gene therapy ↓ bleeding and factor use; liver toxicity reported.
Bolou et al., 2026 [20]	Meta-analysis	720 pediatric patients	Emicizumab → near-zero bleeding; reduced joint damage risk.
Muniz et al., 2023 [21]	Meta-analysis	11 studies	Emicizumab superior to FVIII/BPA; effective in inhibitor patients.

**Abbreviations:** ABR, annualized bleeding rate; QoL, quality of life; FVIII, factor VIII; BPA, bypassing agents; HA, hemophilia A; HB, hemophilia B; NMO, National Member Organization. Symbols: ↑ increase; ↓ decrease; → associated with or leads to.

### 3.2. Impact of Therapeutic Advances

Based on the findings derived from the studies summarized in Table 1, the introduction of advanced therapies, including non-replacement therapies, emicizumab, and gene therapy, has led to a consistent reduction in annualized bleeding rates (ABR), decreased use of clotting factor concentrates, and improvements in quality of life [4,5,18,19,20]. In some populations, particularly pediatric patients treated with emicizumab, bleeding rates approached near-zero levels [20], while gene therapy demonstrated marked reductions in both bleeding events and treatment burden [5,18]. These findings were consistently observed across all included studies evaluating therapeutic advances, including systematic reviews on non-replacement therapies [4], gene therapy [5,18,19], and emicizumab [20,21].

### 3.3. Limitations of Current Therapeutic Strategies

However, these therapeutic benefits are not without limitations. Variability in the durability of gene therapy, including declining factor levels over time and the need for reintroduction of prophylaxis in some patients, has been reported [5,18]. Additional limitations of gene therapy include pre-existing neutralizing antibodies against adeno-associated viral vectors, which may restrict eligibility in a substantial proportion of patients, as well as concerns regarding hepatotoxicity and the need for immunosuppressive management in some cases [5,19]. Moreover, breakthrough bleeding and the lack of long-term data on structural joint outcomes remain relevant concerns [4,14]. In the context of emicizumab, important challenges also persist, including limitations in monitoring with standard coagulation assays and persistent uncertainties regarding the management of specific severe bleeding scenarios. Importantly, several studies also suggest that the introduction of advanced therapies could further widen existing global inequalities due to differences in healthcare systems, access, and affordability [7,17].

### 3.4. Epidemiological Context and Global Disparities in Hemophilia

While the studies identified through the structured search highlighted disparities in access to diagnosis, treatment, and specialized care, complementary epidemiological and contextual evidence provides a broader understanding of the magnitude and distribution of these inequities worldwide.

Data from the World Federation of Hemophilia (WFH) Annual Global Survey 2024 provide a quantitative framework to characterize global disparities in hemophilia identification and care. A total of 271,918 individuals with hemophilia were identified across 135 reporting countries, which is substantially lower than the estimated global burden of approximately 850,000 patients, highlighting significant gaps in detection and reporting [8].

**Figure 2:**
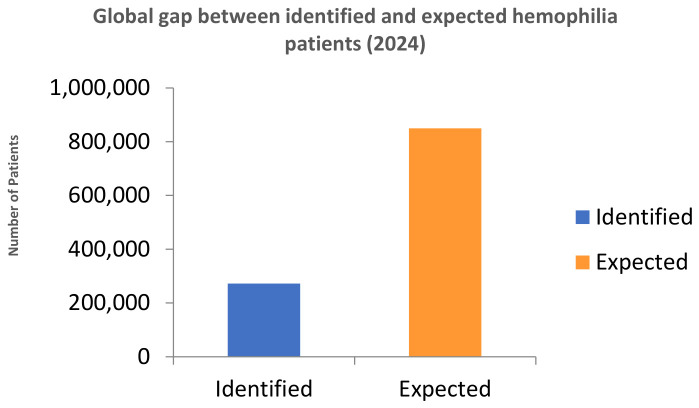
Global comparison between identified and expected hemophilia patients (2024). Expected patient numbers were estimated using a prevalence of 20.9 per 100,000 males. Data adapted from the World Federation of Hemophilia (WFH) Annual Global Survey 2024, using global hemophilia prevalence estimates (p. 3) and patient identification data (p. 12) [8].

Importantly, when these data are interpreted in the context of expected prevalence estimates, a marked discrepancy emerges between identified and expected patients at a global level (Figure 2). This gap reflects substantial underdiagnosis, particularly in low- and middle-income countries, where limitations in diagnostic capacity, registry systems, and healthcare infrastructure remain significant.

Recent literature further supports the existence of substantial structural inequalities in hemophilia care worldwide. Studies focusing on low- and middle-income countries have highlighted persistent barriers related to healthcare infrastructure, availability of diagnostic services, access to comprehensive care programs, and implementation of evidence-based treatment strategies. These challenges continue to limit timely diagnosis and optimal disease management in many regions despite major therapeutic advances [11,22]. Furthermore, international surveys conducted across European countries have demonstrated considerable variability in the organization and delivery of hemophilia care, suggesting that disparities remain evident even within relatively well-resourced healthcare systems [23,24].

**Figure 3:**
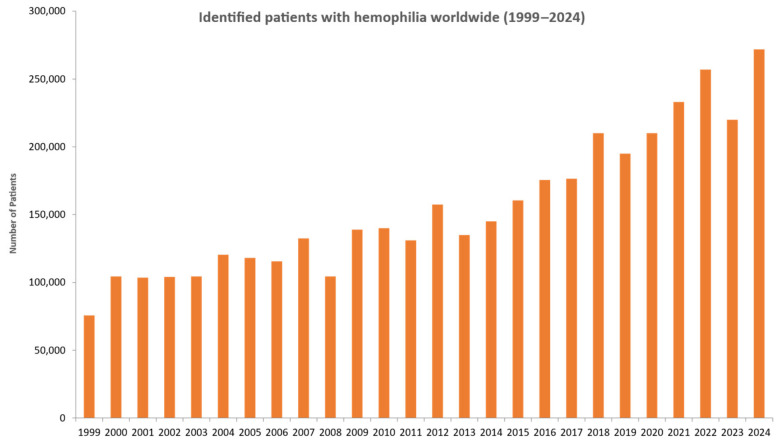
Global trend in the number of identified patients with hemophilia (hemophilia A, B, and type unknown) from 1999 to 2024. Data adapted from the World Federation of Hemophilia (WFH) Annual Global Survey 2024 Interactive Graphs (AGS), using historical hemophilia identification data (Hemophilia A, B, Type Unknown only) [8]. The 2024 value is consistent with the patient identification data reported in the WFH Annual Global Survey 2024 report (p. 12) [8].

Moreover, temporal data from the WFH Annual Global Survey demonstrate a progressive increase in the number of identified patients worldwide from 1999 to 2024, suggesting improvements in awareness, diagnostic strategies, and reporting systems [1,8] (Figure 3). However, this increase is not uniformly distributed across regions, reinforcing the persistence of global inequities in hemophilia care and surveillance.

Importantly, global data also reveal a marked gap between identified and expected patients across regions, highlighting the magnitude of underdiagnosis worldwide. This discrepancy is particularly evident in low- and middle-income regions, where a substantial proportion of individuals with hemophilia remain undiagnosed or outside formal healthcare systems.

**Figure 4:**
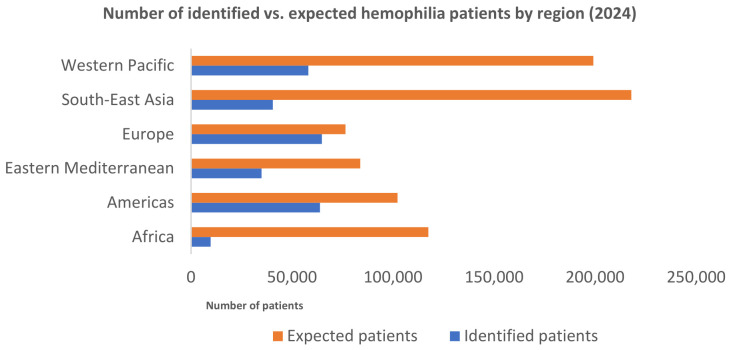
Number of identified versus expected hemophilia patients by region (2024). Data adapted from the World Federation of Hemophilia Annual Global Survey 2024, based on regional patient identification and prevalence data reported (p. 15) [8]. Expected patient numbers were estimated using a prevalence of 20.9 per 100,000 males.

For example, regional analyses indicate that only a small proportion of expected patients are identified in areas such as Africa and Southeast Asia, compared to much higher identification rates in Europe and the Americas, illustrating a profound imbalance in disease recognition (Figure 4).

In addition to disparities in diagnosis and basic care provision, recent evidence suggests that access to innovation is also unevenly distributed. Although advanced therapies have transformed the management of hemophilia in many settings, their implementation remains strongly influenced by economic resources, healthcare policies, reimbursement mechanisms, and local infrastructure. Consequently, substantial differences persist in access to novel treatments, participation in clinical research, and integration of emerging therapeutic approaches across regions [22,25].

Beyond geographical and economic disparities, growing evidence highlights persistent inequities affecting women and girls with bleeding disorders. Historically, hemophilia has been perceived primarily as a male condition, contributing to delayed diagnosis, under-recognition, and reduced access to specialized care among female patients and symptomatic carriers. Recent studies have emphasized the need to improve awareness among healthcare professionals, promote earlier recognition of bleeding symptoms, and increase access to appropriate diagnostic pathways for women and girls [26,27]. Moreover, historical and contemporary analyses indicate that women remain underrepresented in both hemophilia research and clinical trials, potentially limiting the generation of evidence applicable to this population and reinforcing existing disparities in care [28,29].

### 3.5. Global Research Landscape and Contributors in Hemophilia

#### 3.5.1. Evidence from Bibliometric Studies on Research Distribution

Bibliometric analyses in hemophilia remain limited and have primarily focused on specific subfields such as hemophilic arthropathy or broader hematologic disorders, highlighting the need for more comprehensive evaluations of global research activity in hemophilia. A recent bibliometric and visualization analysis based on the Web of Science Core Collection identified the United States as the leading contributor in terms of publications and international collaboration, followed by European countries such as Spain and the Netherlands [10].

At the author level, researchers such as Rodríguez-Merchán have been identified as highly productive contributors, while collaborative networks are predominantly concentrated in North America and Europe [10].

These findings are further supported by co-authorship and institutional analyses, which demonstrate that research activity is highly clustered within a limited number of leading centers, reinforcing the concentration of expertise in specific geographical regions [10].

Complementary bibliometric evidence from gene therapy research in inherited hematologic disorders also confirms the dominant role of the United States, accounting for more than 60% of publications, alongside strong international collaboration networks involving European countries [30].

This consistency across distinct research areas suggests that the observed geographical concentration is not domain-specific but reflects broader structural determinants of scientific production.

Additional bibliometric evidence focused on haemophilia-related pain further supports this geographical pattern, with the United States leading scientific production, followed by Germany, Spain, and the United Kingdom, suggesting consistency across different research domains within hemophilia [31].

Notably, this pattern persists despite differences in research focus, indicating a stable and reproducible distribution of scientific activity across subfields.

Taken together, these findings indicate that the geographical concentration of research output is consistent across different domains within hemophilia, reinforcing the robustness and reproducibility of this pattern.

These findings highlight a clear geographical imbalance in research production, with high-income countries leading scientific output, while other regions remain underrepresented.

#### 3.5.2. Integration with Global Epidemiological Data

Data from the WFH Annual Global Survey show marked global disparities in the identification of hemophilia cases, with higher rates of identified patients per 100,000 inhabitants in high-income countries compared to low- and middle-income regions [1,6,8].

This pattern is consistent with the geographical distribution of research output identified in bibliometric analyses, suggesting that scientific production is closely linked to healthcare infrastructure, treatment availability, and the existence of national registries.

Moreover, WFH data also reveal substantial differences in access to clotting factor concentrates and specialized hemophilia treatment centers (HTCs), with markedly lower availability in low-resource settings [6,7,8]. These structural differences further contribute to the observed imbalance in both clinical outcomes and research productivity.

Importantly, these epidemiological disparities mirror the distribution of research output, suggesting a bidirectional relationship between healthcare capacity and knowledge generation [32].

#### 3.5.3. Global Disparities and Structural Determinants

The available evidence consistently indicates a predominance of research activity in high-income countries, particularly the United States and several European nations [10,30,31]. This pattern aligns with regions showing higher diagnostic rates, access to treatment, and established research infrastructures [6,7,8,10].

In contrast, bibliometric and epidemiological evidence suggests substantially lower research representation in low- and middle-income regions. This likely reflects structural barriers, including a lack of research infrastructure, the absence of national registries, limited access to comprehensive care, and limited availability of treatment [6,8,30,32].

These disparities suggest a structural relationship between healthcare capacity and research output, reinforcing the concept of a global imbalance not only in care delivery but also in knowledge generation [32].

Global estimates further suggest that a significant proportion of individuals with hemophilia worldwide remain undiagnosed or inadequately treated, particularly in low-resource settings, which limits the generation of high-quality epidemiological and clinical research data [6,8].

#### 3.5.4. Musculoskeletal Research Focus

From a musculoskeletal perspective, hemophilic arthropathy remains one of the main sources of morbidity, even in settings with access to replacement therapy [13].

Despite its clinical relevance, bibliometric analyses suggest that musculoskeletal research in hemophilia is concentrated in a relatively limited number of research groups and institutions, reflecting a potential gap between clinical burden and research prioritization [10].

#### 3.5.5. Future Challenges and Research Sustainability

Concerns regarding the future direction and sustainability of research priorities in hemophilia have previously been discussed in the literature. As early as 2006, Srivastava raised concerns that major therapeutic advances could potentially influence long-term research engagement in the field [33].

The rapid evolution of advanced therapies may also reshape future research priorities in hemophilia. Despite major improvements in hematologic control, persistent unmet needs—including musculoskeletal complications, long-term functional outcomes, and real-world implementation challenges—remain clinically relevant and continue to require sustained research attention [14,34,35].

#### 3.5.6. Final Synthesis

Overall, the global landscape of hemophilia research reflects a clear geographical and structural imbalance. Scientific production is concentrated in high-income countries with established healthcare systems, while low-resource settings remain underrepresented both in clinical care and research output [8,10,30].

These findings collectively highlight the need for more geographically inclusive research strategies aimed at strengthening research capacity, improving epidemiological surveillance, and expanding data generation in underrepresented regions.

This imbalance further reinforces the existence of a global mismatch between therapeutic advances and their real-world implementation, emphasizing the importance of equitable access to care, international collaboration, and context-adapted healthcare strategies.

## 4. Discussion

The present review highlights a clear geographic and structural imbalance in hemophilia research and care. The majority of scientific output originates from high-income countries with well-developed healthcare systems, such as the United States, Canada, and Western Europe. These regions benefit from integrated academic-clinical networks, national registries, and access to advanced therapies, which facilitate high-quality research production and implementation [3,10].

However, this concentration of knowledge raises important concerns regarding the generalizability of current evidence to under-resourced populations. Much of the available literature is derived from populations with early diagnosis, access to prophylaxis, and multidisciplinary care. In contrast, patients in low- and middle-income countries (LMICs) often present with delayed diagnosis, limited access to treatment, and advanced joint disease, conditions that are underrepresented in the scientific literature [9,32]. Importantly, disparities in hemophilia care are not restricted to comparisons between high- and low-income regions. Surveys conducted across European countries have demonstrated substantial heterogeneity in factor consumption, access to prophylaxis, home treatment programs, and organization of care, highlighting the persistence of unmet needs even within economically developed healthcare systems [23,24]. These findings suggest that healthcare organizations, national policies, registry development, availability of specialized treatment centers, and long-term investment in hemophilia care may be as important as economic resources themselves in determining patient outcomes and access to treatment.

Second, low- and middle-income countries remain significantly underrepresented in the scientific literature. This underrepresentation is not only a reflection of limited research capacity, but also contributes to a cycle of invisibility, where the specific needs and realities of these populations are not adequately addressed [7]. Persistent gaps in diagnosis, treatment availability, and healthcare infrastructure continue to limit the translation of scientific advances into routine clinical practice in many regions of the world [6,7]. Consequently, disparities in care and disparities in knowledge generation appear to be closely interconnected phenomena rather than independent challenges.

The findings of this review confirm that global disparities in hemophilia extend beyond access to treatment and include structural limitations in healthcare systems, diagnostic capacity, and research infrastructure. These inequalities are further exacerbated in specific populations, such as women and girls with bleeding disorders, who remain underdiagnosed and undertreated even in high-income settings [15,16]. Recent international surveys have further documented persistent barriers affecting women and girls with bleeding disorders, including limited awareness among healthcare professionals, lack of dedicated services, diagnostic delays, socioeconomic constraints, and underrepresentation in decision-making structures, all of which continue to restrict equitable access to care [9]. These findings are consistent with broader public health analyses indicating that women and girls with bleeding disorders continue to experience delayed diagnosis, under-recognition of symptoms, and barriers to specialized care pathways despite growing awareness of their clinical needs [26].

In parallel, the introduction of advanced therapies has profoundly transformed the clinical landscape of hemophilia. Systematic reviews and meta-analyses consistently demonstrate significant reductions in bleeding rates, decreased use of clotting factor concentrates, and improvements in quality of life across different therapeutic approaches, including non-replacement therapies, emicizumab, and gene therapy [4,5,18,20,21].

However, despite these advances, several studies highlight important limitations that challenge the perception of a fully resolved disease. Variability in the durability of gene therapy, the persistence of breakthrough bleeding, and the lack of long-term data on structural joint outcomes remain significant concerns [14,19]. Importantly, modelling studies suggest that the impact of these therapies on health equity is highly dependent on healthcare systems and funding decisions, indicating that therapeutic innovation alone is insufficient to address global disparities [17]. This observation is consistent with previous analyses emphasizing that therapeutic innovation does not automatically translate into equitable access. The persistence of diagnostic gaps, treatment shortages, and infrastructure limitations in many countries highlights the need for parallel investments in healthcare systems, workforce development, and implementation capacity if the benefits of the current therapeutic revolution are to be distributed more evenly worldwide [6,7]. This consideration also extends to safety-related aspects and their potential inequalities, as historical concerns regarding pathogen transmission have influenced the evolution of hemophilia therapies [1].

From a musculoskeletal perspective, the persistence of hemophilic arthropathy represents one of the most critical unresolved challenges. Several studies demonstrate that, despite reductions in bleeding frequency, structural joint damage may persist or progress. Longitudinal and real-world data show that extended half-life therapies reduce hemarthrosis and pain but do not significantly improve joint health parameters such as range of motion, muscle strength, or joint scores [34]. Similarly, imaging studies reveal the presence of subclinical joint damage even in patients with excellent clinical control [35].

This discrepancy between clinical improvement and structural outcomes is increasingly explained by the role of subclinical bleeding. Emerging evidence indicates that joint damage may occur in the absence of clinically evident bleeding, highlighting the limitations of traditional outcome measures such as the annualized bleeding rate (ABR) [12,36]. In fact, recent studies demonstrate that laboratory parameters, including clotting factor levels or global coagulation assays, are poor predictors of bleeding risk, whereas joint status assessed by imaging is more closely associated with clinical outcomes.

In this context, musculoskeletal ultrasound (MSK-US), particularly point-of-care ultrasound (POCUS), may represent a useful complementary tool for the evaluation and monitoring of hemophilic arthropathy. Ultrasound allows early detection of synovitis, joint effusion, and structural changes, offering a rapid, lower-cost, and repeatable complement to magnetic resonance imaging (MRI) in clinical practice [13]. Additionally, prospective real-world studies have suggested that systematic joint assessment using ultrasound may influence clinical decision-making, including treatment adjustments and rehabilitation strategies [37].

However, it is important to recognize that ultrasound has limitations, particularly in the evaluation of deep osteochondral structures, and should be considered complementary rather than a replacement for MRI. Additionally, the absence of standardized protocols and operator dependency remain challenges for widespread implementation [12].

Taken together, these findings support the concept that modern hemophilia management requires a shift from a predominantly hematological perspective toward a more comprehensive and multidisciplinary approach [12,13].

In this context, the gap in care extends beyond pharmacological treatment. While the lack of access to clotting factor concentrates is well documented, the absence of structured rehabilitation programs has received comparatively little attention. Musculoskeletal involvement continues to represent a leading contributor to long-term functional impairment, and its management requires long-term, multidisciplinary approaches [11,13].

Physiotherapy may therefore represent a relevant complementary intervention within comprehensive hemophilia care. Rehabilitation-based strategies may contribute to functional preservation and long-term musculoskeletal management across different healthcare settings. In settings where access to advanced therapies is limited, physiotherapy may play an important supportive role within multidisciplinary care models. Preventive strategies, functional rehabilitation, and patient education may contribute to improving functional outcomes and quality of life, although important implementation barriers remain in many resource-limited settings. Importantly, disparities in access to rehabilitation services may mirror the broader inequalities observed in hemophilia care. In many low-resource settings, limited availability of trained physiotherapists, specialized rehabilitation programs, and multidisciplinary teams may further restrict opportunities for long-term musculoskeletal management, even when pharmacological treatment is available [11].

Moreover, simplified diagnostic approaches—such as focused musculoskeletal assessment protocols—may contribute to earlier detection and longitudinal monitoring of joint involvement, although their implementation may vary depending on local healthcare resources and infrastructure.

These findings call for a redefinition of current care models, particularly in resource-limited settings. Instead of relying exclusively on high-cost pharmacological solutions, there is a need to integrate low-cost, accessible interventions into standard care models. This consideration is particularly relevant in LMICs, where healthcare systems frequently face competing priorities and limited resources. In these contexts, feasible and accessible interventions may provide opportunities to improve patient outcomes while reducing dependence on highly specialized infrastructure [11,22].

Future research should focus on developing and validating context-adapted strategies, including physiotherapy protocols and simplified diagnostic tools, to improve care in underserved populations. Future studies should also evaluate the real-world feasibility, implementation barriers, and cost-effectiveness of these strategies across different healthcare settings, particularly in low- and middle-income countries.

Importantly, this imbalance may also have implications for the long-term sustainability of research in hemophilia, as previously suggested [33], where concerns were raised regarding declining engagement of new investigators and the perception of reduced clinical challenges in well-resourced settings.

Similarly, historical analyses of hemostasis research development highlight how research activity is strongly conditioned by local infrastructure and resource availability [38], reinforcing the structural nature of global disparities in both care and knowledge production.

Importantly, addressing global disparities in hemophilia care should not rely exclusively on expanding access to high-cost pharmacological therapies. Complementary strategies, including physiotherapy, patient education, and simplified diagnostic tools, may provide realistic and impactful alternatives in low-resource environments. Achieving a more equitable paradigm shift will therefore require not only therapeutic innovation, but also sustained investment in healthcare systems, workforce development, and implementation capacity across diverse healthcare settings [6,7].

### Limitations

This study has several limitations. First, the selection of studies was based on a structured but non-systematic search strategy, which may introduce selection bias and limit the comprehensiveness of the review. Although a structured workflow was used to improve transparency regarding the literature identification and selection process, this review was not designed as a formal systematic review and therefore did not include methodological components such as risk of bias assessment or quantitative synthesis. Furthermore, the literature search was intended to support and contextualize the narrative framework of the review rather than to provide an exhaustive systematic assessment of all available evidence.

Second, the discussion of global research distribution was based on published bibliometric studies and should not be interpreted as a formal bibliometric analysis performed by the authors. In addition, some complementary references incorporated to contextualize global disparities, healthcare inequities, and research distribution were not identified through the structured search strategy but were included to support the narrative interpretation of the findings.

Finally, the heterogeneity of the included studies, particularly in terms of design, populations, and outcome measures, limits the ability to perform direct comparisons or draw definitive conclusions.

## 5. Conclusions

Hemophilia care has undergone a profound transformation with the advent of advanced therapies, including extended half-life products, non-replacement therapies, and gene therapy, which have significantly improved bleeding control and patient outcomes. However, these therapeutic advances have not been translated uniformly across different healthcare settings, resulting in persistent global disparities in access to care, healthcare infrastructure, research capacity, and clinical outcomes.

The findings of this study highlight a clear mismatch between the rapid evolution of therapeutic options and their real-world implementation, particularly in low- and middle-income countries, where limitations in healthcare infrastructure, diagnostic capacity, and access to treatment remain substantial. In this context, the concentration of scientific evidence in high-income settings further limits the generalizability of current knowledge to under-resourced populations.

Importantly, improved hematologic control does not necessarily equate to preservation of musculoskeletal health. Structural joint damage, subclinical bleeding, and persistent arthropathy remain clinically relevant challenges, reinforcing the need for systematic structural monitoring as a complement to pharmacological management.

In this scenario, physiotherapy and rehabilitation may represent important complementary components of comprehensive hemophilia care. Beyond their traditional role in post-bleeding recovery, these interventions may contribute to prevention, functional preservation, and patient education, particularly as lower-cost complementary approaches within multidisciplinary care models. Similarly, simplified diagnostic approaches, including musculoskeletal ultrasound, may represent useful complementary strategies for early detection and longitudinal monitoring of joint involvement, particularly in settings where access to advanced imaging modalities is limited, although their implementation may vary depending on local healthcare resources and infrastructure.

Addressing global disparities in hemophilia care requires a shift toward more inclusive and adaptable care models that integrate advanced therapies with low-cost, widely implementable strategies. The development of multidisciplinary approaches, supported by standardized assessment tools and structural monitoring, represents a key step toward achieving equitable and sustainable care.

Future efforts should focus on strengthening research capacity in underrepresented regions, validating context-specific clinical strategies, and promoting global collaboration to ensure that therapeutic advances translate into meaningful improvements in joint health, function, and quality of life for all individuals with hemophilia. Reducing the global gap in hemophilia care will require not only continued therapeutic innovation but also sustained investment in healthcare infrastructure, implementation capacity, and equitable access to multidisciplinary care.

## Data Availability

No new data were created or analyzed in this study. Data sharing is not applicable.
